# Supplementary material for: Gene Expression Changes in Cytokine and Chemokine Receptors in Association with Melanoma Liver Metastasis
Source: Int J Mol Sci. 2023 May 17;24(10):8901. doi: 10.3390/ijms24108901 (PMC10219520; doi:10.3390/ijms24108901)
Supplement: Supplementary file 1 [file ijms-24-08901-s001.zip › Koroknai_etal_Supplementary Table S1.pdf]

Supplementary Table S1. Results of *in vitro* invasion assays

	WM983A	WM278	WM3248	WM793B	WM1361	WM1366
Control[a] 1	4.3	321	1.0	312	12.7	384
Control 2	7.9	314	8.5	301	18.8	365
Control 3	5	99	1.3	320	36	372
HHSEC-CM[b] 1	9.4	301	7.3	314	20.8	352
HHSEC-CM 2	11.3	325	27.0	305	35.1	368
HHSEC-CM 3	9.1	114	6.9	339	63	367
Ratio[c] 1	2.2	0.9	7.3	1.0	1.6	0.9
Ratio 2	1.4	1.0	3.2	1.0	1.9	1.0
Ratio 3	1.8	1.2	5.3	1.1	1.8	1.0
Mean ratio	1.8	1.0	5.3	1.0	1.8	1.0
S.D. ratio	0.4	0.1	2.1	0.0	0.1	0.0

[a]Average number of invaded cells with 10% FBS as chemoattractant, [b]Average number of invaded cell with HHSEC conditioned medium (50%) as chemoattractant. [c]Ratio of the HHSEC-CM and Control results, numbers indicate the same experiments.
