# Supplementary material for: Gene Expression Changes in Cytokine and Chemokine Receptors in Association with Melanoma Liver Metastasis
Source: Int J Mol Sci. 2023 May 17;24(10):8901. doi: 10.3390/ijms24108901 (PMC10219520; doi:10.3390/ijms24108901)
Supplement: Supplementary file 1 [file ijms-24-08901-s001.zip › Koroknai_etal_Supplementary Table S3.pdf]

Supplementary Table S2. Correlation between the relative gene expression of cytokine- and chemokine receptors and the invasive potential in melanoma cells after culturing with HHSEC-CM.

Gene symbol	Correlation Coefficient	Sig. (2-tailed)
CCR1	.899*	0.015
CXC3CR1	.896*	0.016
CXCR2	.870*	0.024
CXCR5	.877*	0.022
IL1RAPL2	.963**	0.002
IL1RN	.928**	0.008
IL6ST	.919**	0.010
IL15RA	.933**	0.007
IL17RB	.967**	0.002
IL17RC	.872*	0.024
IL17RD	.938**	0.006
TNFRSF10B	.918**	0.010
TNFRSF18	.925**	0.008

* Correlation is significant at the 0.05 level (2-tailed).

** Correlation is significant at the 0.01 level (2-tailed).
