# Supplementary figures and images for: Gene Expression Changes in Cytokine and Chemokine Receptors in Association with Melanoma Liver Metastasis
Source: Int J Mol Sci. 2023 May 17;24(10):8901. doi: 10.3390/ijms24108901 (PMC10219520; doi:10.3390/ijms24108901)

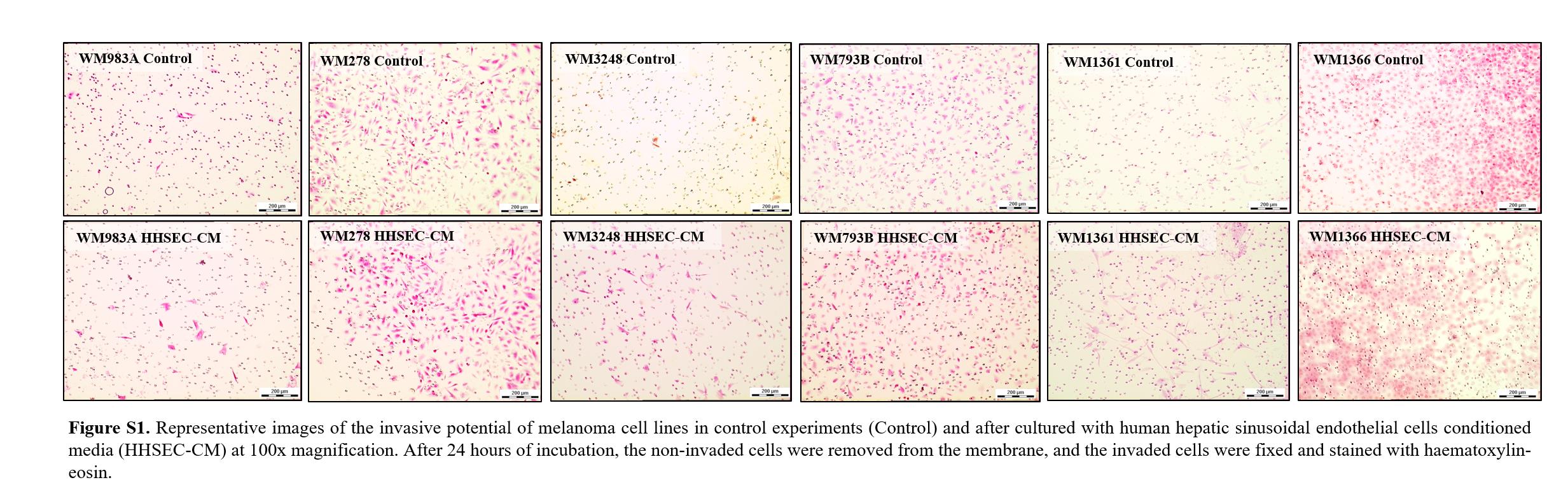

Supplement: Supplementary file 1 [file ijms-24-08901-s001.zip › Supplementary Figure S1_NEW.jpg]
